# SER: An R package to characterize environmental regimes

**DOI:** 10.1002/ece3.9882

**Published:** 2023-03-12

**Authors:** Naicheng Wu, Kun Guo, Yi Zou, Fengzhi He, Tenna Riis

**Affiliations:** ^1^ Department of Geography and Spatial Information Techniques Ningbo University Ningbo China; ^2^ School of Ecological and Environmental Sciences East China Normal University Shanghai China; ^3^ Department of Health and Environmental Sciences Xi'an Jiaotong‐Liverpool University Suzhou China; ^4^ Leibniz Institute of Freshwater Ecology and Inland Fisheries Berlin Germany; ^5^ Department of Biology Aarhus University Aarhus C Denmark

**Keywords:** environmental legacy, environmental variables, historical legacy, R package, regime, SER

## Abstract

Environmental regimes (or environmental legacy or historical legacy) are the dynamics of environmental characteristics over a given (either long or short) time period, such as frequency of mean or extreme events and rate of change, which might be absent by using only contemporary variables. We present SER, an R package for estimating environmental regimes for different environmental variables. Using the data included in the package, several examples are shown. SER is suitable for any type of environmental or biotic variables, including nutrient concentration, light, and dissolved oxygen. In addition, by changing the argument “days_bf,” it is possible to compute environmental regimes over any time period, such as days, months, or years. Our case study showed that the inclusion of environmental regimes increased the explained variation of temporal β‐diversity and its components. Environmental regimes are expected to advance the “environment–community” relationships in ecological studies. They can further be implemented in other subjects such as social science, socioeconomics, and epidemiology.

## Graphical abstract

We present a new R package to compute environmental regimes over a certain period, which has important implications for basic and applied ecology, as well as other subjects.

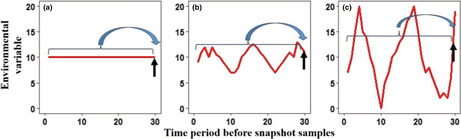

## 1 INTRODUCTION

**FIGURE 1:**
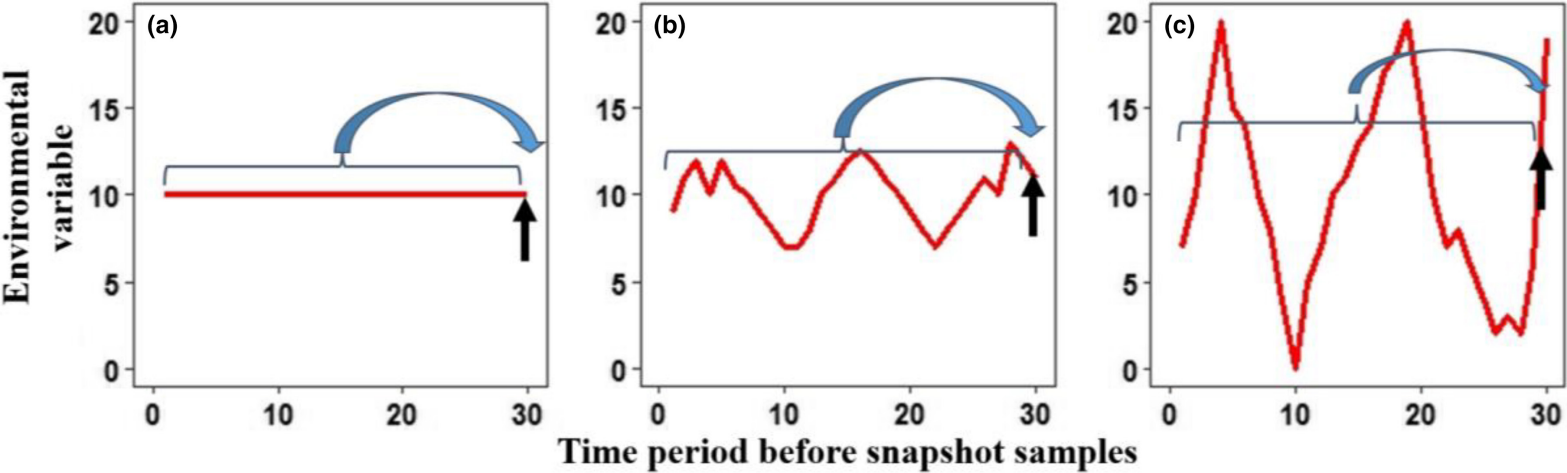
Schematic figure showing 3 scenarios of environmental conditions over a certain time period (within curly brackets) before snapshot samples (indicated by black arrows). Scenario a: environmental variable being constant; b: weak fluctuations in the environmental variable; and c: strong fluctuations in the environmental variable over the whole time period (i.e., from time 0–30). Commonly used indices such as instant value, simple mean, or median values do not sufficiently represent the environment regimes/fluctuations prior to the sampling date in scenarios b and c.

A sound understanding of environment–community relationships is a central topic in ecology. Scientists have been endeavoring to find suitable environmental variables or indices that have potential impacts on community compositions and distributions. Traditionally, snapshot contemporary environmental variables that were collected simultaneously with biological samples, such as in situ parameters and nutrient concentrations, are often employed. However, such snapshot neglects the fact that the biological community responds not only to contemporary environmental conditions but also to historic environmental (also called historic legacy) characteristics (Figure 1) (Su et al., 2022). For example, Oliveira et al. (2020) found that current environmental variables were weak predictors of fish community structure, but the predictive power substantially increased when using dataset obtained in a previous time period. In response, new indices that integrate long‐term environmental records were proposed. For instance, hydrologic indicators for characterizing streamflow regimes (i.e., flow regimes) using long‐term flow records have been developed to represent biologically relevant streamflow attributes (Olden & Poff, 2003). Another example is the 19 standard bioclimatic indices, which integrate climate data from 1970 to 2000 (available in WorldClim 2 database; Fick & Hijmans, 2017). In addition, historical legacies (i.e., past climate and geography: temperature anomaly during the quaternary period, past temperature trend, past precipitation trend, past climate‐change velocity, basin median latitude, and the endorheic/exorheic status of the river) were computed and used to explore their roles in shaping functional diversity of global freshwater fishes (Su et al., 2022). The results showed that the historical legacies significantly imprinted the functional dispersion and functional identity patterns.

The utility of these historical environmental regime indices (e.g., hydrologic indicators, bioclimatic indices) has resulted in tremendous applications in ecological research and also other lines of research (e.g., De Pauw et al., 2022; Nguyen et al., 2021; Su et al., 2022; Tonkin et al., 2018; Tornés et al., 2021; Xu et al., 2022). However, there are several constraints to the currently used historical environmental regime indices: (1) The currently available indices are limited to hydroclimatic variables, such as flow, temperature, and precipitation. There is no available R package to integrate all environmental variables with consideration of any biotic and abiotic factors such as pH, turbidity, dissolved oxygen, and chlorophyll a; (2) these aforementioned indices are mostly based on long‐term intervals, e.g., 30 years for bioclimatic variables. Given that some organisms, particularly microorganisms, may show quick responses to environmental changes, the aforementioned indices might fail to link with biotic changes, and a shorter time period may be more relevant. In addition, different organisms (e.g., algae, macroinvertebrates, fish, macrophytes, or even terrestrial plants) have a distinct extent of response to historical environmental regimes. For instance, recent studies found that flow regimes over a short‐term period (e.g., 7 or 14 days) played a vital role in riverine algae and biofilm communities (Guo et al., 2020; Guo et al., 2021; Qu et al., 2019; Wu et al., 2018). By contrast, macroinvertebrate and fish communities may show a good response to environmental changes over a longer time period (e.g., 4 weeks, 1 year) (Schneider & Petrin, 2017). Therefore, to differentiate the distinct responses of different organisms, we should derive community‐specific indices that describe environmental patterns over relevant time periods. Unfortunately, no R package so far provides a function to calculate indices over a required time period.

Prompted by the importance of environmental characteristics over a certain time period and their research scarcity in this field, we here propose a new term for “environmental regime” (or environmental legacy or historical legacy). Unlike the traditional environmental variables, these new environmental regime indices are defined as the environmental dynamic characteristics during a given (either long or short) time period, which might be absent by using contemporary environmental variables or simple average or median values (Figure 1). With the facilitation of science and technology, high resolution (measured by daily, sub‐daily, hourly, or even finer scale) environmental variables (e.g., nutrient concentration, dissolved oxygen) are available nowadays. An increasing number of studies have used data from high‐frequency measurements, e.g., water temperature and dissolved oxygen in lakes (Meinson et al., 2015) or soil greenhouse gas fluxes (Courtois et al., 2019). These data provide scientists a chance to explore research questions at time scales that were not possible earlier. Further, high‐frequency data allow computing environmental regimes that can be potential variables to increase the explained variation of biological communities (e.g., Guo et al., 2020; Wijewardene, Wu, Giménez‐Grau, et al., 2021; Wu et al., 2019). Therefore, this study aims to facilitate the computation of those indices by providing a common and extensible platform.

## 2 THE SER PACKAGE: SHORT‐PERIOD ENVIRONMENTAL REGIME

The SER package aims to compute environmental regimes over a certain time period. In total, 11 elementary indices that focus on variations of environmental factors over a given short period were developed (Table 1). These indices, inspired by Olden and Poff (2003), elucidate three aspects, i.e., the magnitude, the frequency, and the rate of change of environmental variables over a given time period. The magnitude contains four indices: mean, median, coefficient of variation, and skewness of the variables over a given time period before the snapshot sampling; frequency demonstrates the number of environmental low or high pulses over a given time period before the snapshot sampling; rate of change how fast the environmental variable changed (i.e., positive or negative change) within the given time period before the snapshot sampling.

**TABLE 1:** Definition of the 11 elementary indices for environmental regimes.

Code	Unit	Definition
Magnitude of environmental data		
MA1	Depends	Mean of the environmental variable over a given time period (*N*)
MA2	Depends	Median of the environmental variable over a given time period (*N*)
MA3	%	Coefficient of variation over a given time period (*N*)
MA4	–	Skewness of the environmental variable for a given time period (*N*)
Frequency of environmental data		
ML1	Temporal unit	Number of low pulse events over a given time period (N): numbers of occurrences where the magnitude of an environmental variable drops below the 25th percentile of all values for the given time period
MH1	Temporal unit	Number of high pulse events over a given time period (*N*): numbers of occurrences where the magnitude of an environmental variable is above the 75th percentile of all values for the given time period
EL1	Temporal unit	Number of extreme low pulse events over a given time period (*N*): numbers of occurrences where the magnitude of an environmental variable drops below the 10th percentile of all values for the time period
EH1	Temporal unit	Number of extreme high pulse events over a given time period (*N*): numbers of occurrences where the magnitude of an environmental variable is above the 90th percentile of all values for the time period
Rate of change of environmental data		
RC	Depends	Rate of change over a given time period (*N*)
RH1	Temporal unit	Numbers of events with rising change over a given time period (*N*), i.e., numbers of positive change
RL1	Temporal unit	Numbers of events with declining change over a given time period (*N*), i.e., numbers of negative change

*Note*: Depends: the unit depends on the variables; −: no unit; Temporal unit: the given temporal units, e.g., days, months, or years. *N* can be defined as any interested time period, by default N = BetwSamT (days between two successive sampling dates). Currently, the package is developed with regarding days, months, and years as the interested period, more features are under development. To be consistent with a previous study (Olden & Poff, 2003), we choose 25th/10th and 75th/90th percentile as low/extreme low and high/extreme high values for the frequency indices.

### 2.1 Package overview

The SER package contains one main function *SER* and two data files, i.e., *hydro_df* and *sample_date*. The two data files are derived from Guo et al. (2020) and are used to illustrate how the main function works. The *hydro_df* is a data frame that contains daily discharge in a stream, while *sample_date* is a vector containing 13 dates, first of which is the date when the experiment was initialized while the rest 12 are snapshot biological sampling dates.

The SER package is written in R (R Core Team, 2021) and require a standard installation of R and the “tidyverse” and “lubridate” packages. The development version of SER is hosted on GitHub at https://github.com/kun‐ecology/SER. The package can be installed with the following codes:
if(!require(devtools))install.packages(“devtools”).


devtools::install_github(“https://github.com/kun‐ecology/SER,” build_vignettes = TRUE).



### 2.2 Example analyses

As an example, the embedded data are used to illustrate how SER works with discharge data. By default, days between two successive sampling dates were used as the focal short period. The R codes are shown below:
# load required packages
library(SER)
# inspect the discharge data
str(hydro_df)
# inspect the sample dates
str(sample_date)
# calculate short‐period hydrological indices
hydro_ser<−SER(hydro_df$Date, hydro_df$Discharge, sample_date, days_bf = NULL)
str(hydro_ser)



**FIGURE 2:**
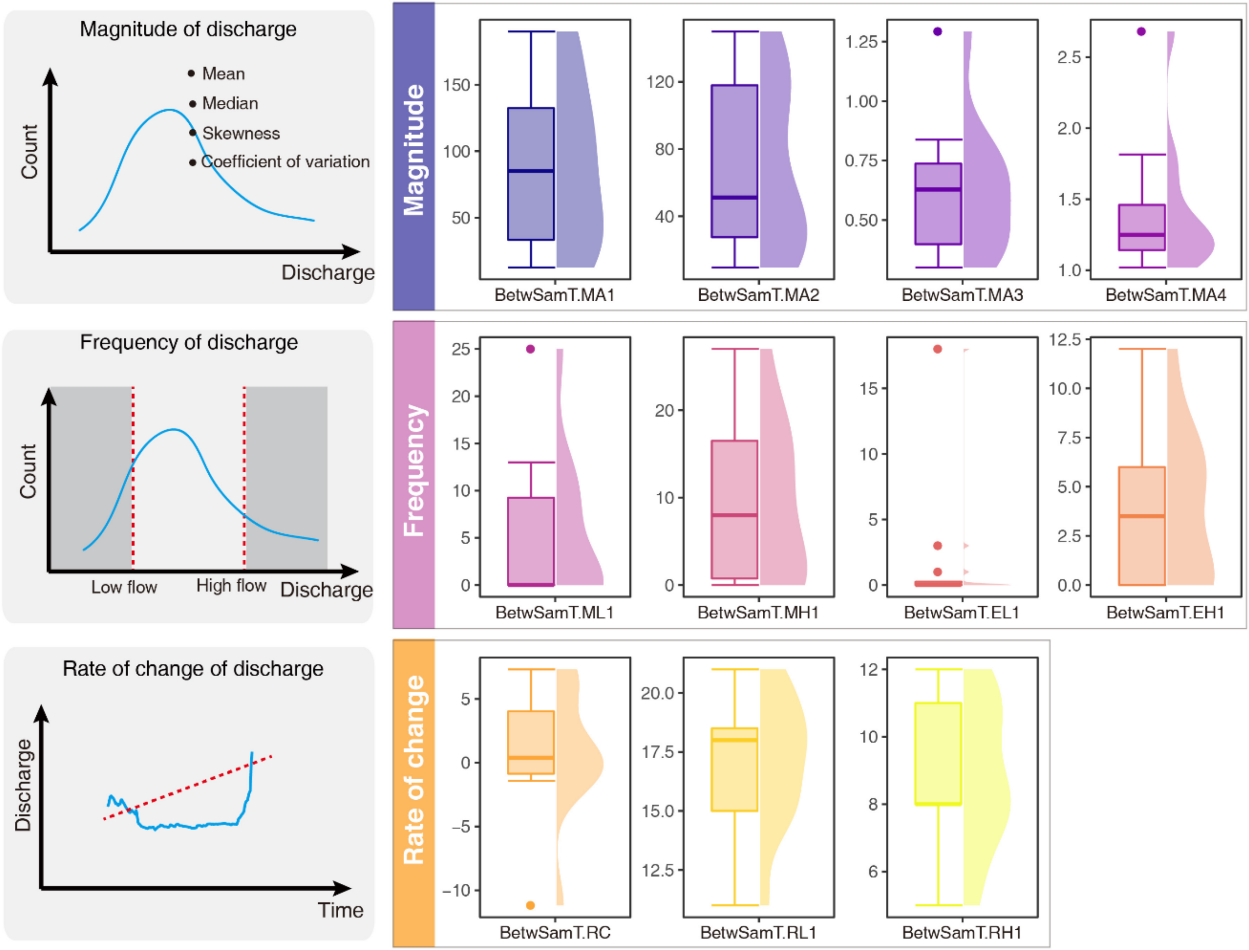
Boxplots (median, first, and third quantiles) and violin plots illustrate the distribution of 11 short‐period hydrological indices calculated in the example of SER package. These indices decipher the three facets, i.e., magnitude, frequency, and rate of change, of flow regime over the time between two successive samplings. See Table S1 for the definition of each index. Indices generated with new data or new time period should be interpreted with a full understanding of the 11 elementary indices and expertise in a given area.

The function SER returned a data frame, in which, the first column “SampleDate” contains the 12 biological sampling dates, while the rest 11 columns represent the short‐period environmental regimes, i.e., short‐period hydrological indices for each sampling date (Figure 2). The indices' names were constructed as the combination of short period and names of the elementary indices, for example, BetwSamT.MA1 and BetwSamT.RC stand for the mean of the daily average flow and mean rate of change in days between two successive sampling days, respectively.

## 3 A CASE STUDY: ENVIRONMENTAL REGIMES PLAY AN IMPORTANT ROLE IN TAXONOMIC AND FUNCTIONAL TEMPORAL β‐DIVERSITY OF RIVERINE DIATOMS

To examine whether the inclusion of environmental regimes advances our understanding of environment‐biota relationships, daily samples of riverine diatom communities over a 1‐year period were collected at a German lowland catchment (Wu, Wang, et al., 2022). Concurrently, three categories of abiotic factors were obtained: (a) hydrological variables (Hyd) included daily discharge (Q), discharge skewness (Sk), precipitation (Prec), and antecedent precipitation index (API); (b) metal ions (Met) contained six parameters (Cl^−^, K^+^, Ca^+^, Na^+^, Mg^2+^, and Si^2+^); and (c) nutrients (Nut) included ammonium‐nitrogen (NH_4_‐N), nitrate‐nitrogen (NO_3_‐N), orthophosphate (PO_4_‐P), and sulphate (SO_4_^2−^). In addition, environmental regimes of both flow (using Q) and nutrient (using NH_4_‐N, NO_3_‐N, PO_4_‐P, and SO_4_^2−^) were computed with *SER* package. Therefore, we have two extra abiotic factors: Hyd + (i.e., hydrology + flow regimes) and Nut+ (i.e., nutrient + nutrient regimes) (Wu, Wang, et al., 2022). Furthermore, both taxonomic and functional temporal β‐diversity of riverine diatoms were computed (for details see Wu, Wang, et al., 2022).

**FIGURE 3:**
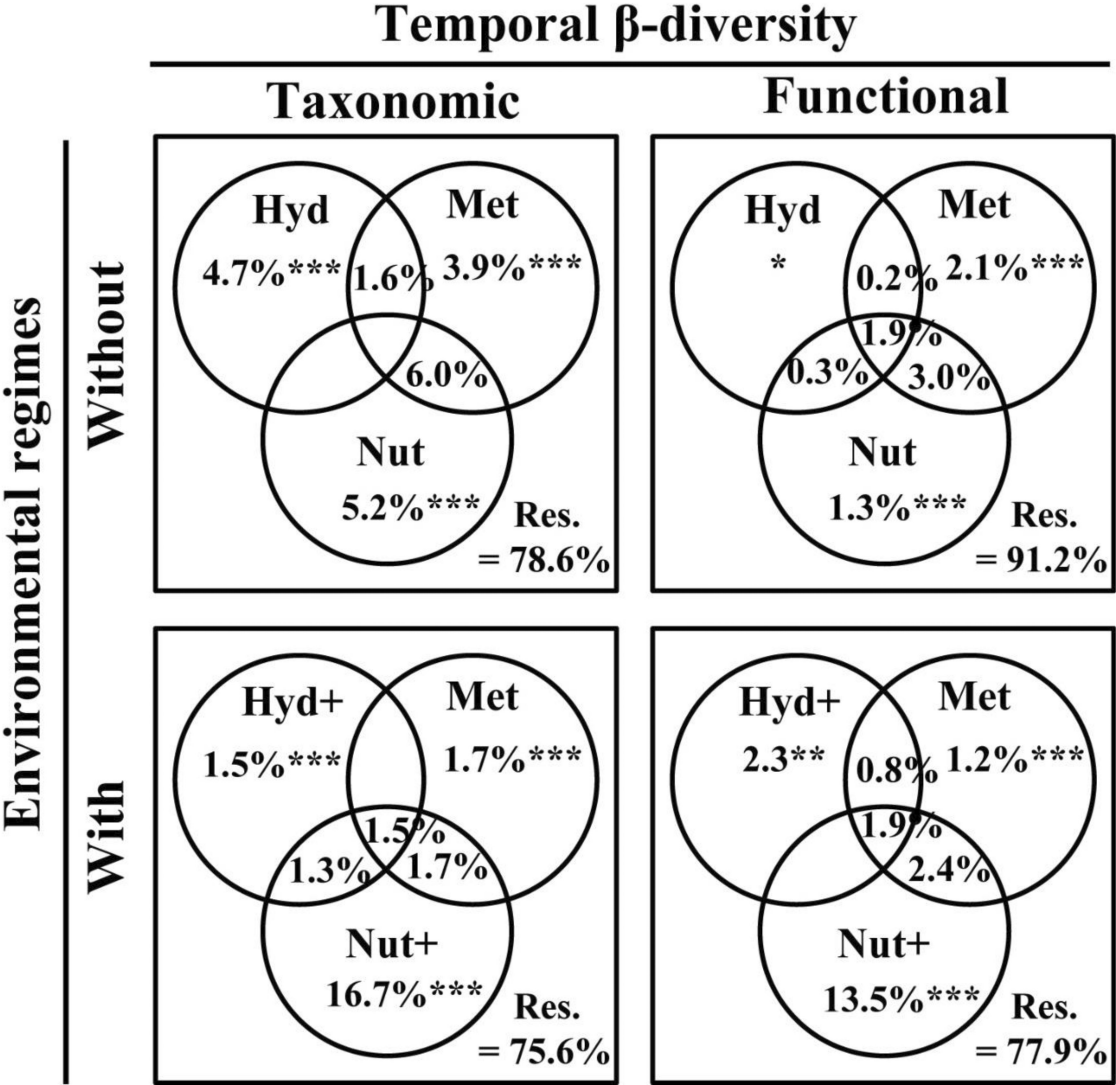
Comparison (between without and with environmental regimes) of the explained variations to taxonomic and functional temporal β‐diversity of riverine diatoms. Hyd, hydrology without flow regimes; Met, metal ions; Nut, nutrients; Hyd+, hydrology with flow regimes; Nut+, nutrients with nutrient regimes. The adjusted *R* ^2^ is shown. ***p < .001, **p < .01, *p < .05. The figure was modified from Wu, Wang, et al. (2022).

Using distance‐based redundancy analysis (db‐RDA; with *capscale* function in R package *vegan*) (Oksanen et al., 2019) and variation partitioning analysis (VPA; with *varpart* function in R package *vegan*), we investigated the relationships between abiotic factors and temporal β‐diversity of riverine diatoms (for details see Wu, Wang, et al., 2022). To detect the role of environmental regimes in explaining the variation of both taxonomic and functional temporal β‐diversity, we compared the explained variations between without and with environmental regimes. VPA results demonstrated that the addition of environmental regimes (i.e., flow and nutrient regimes) increased the explained variations of both taxonomic and functional temporal β‐diversity (Figure 3). Specifically, taxonomic total β‐diversity increased by 3.0%, while functional total β‐diversity increased by 13.3%. Interestingly, the inclusion of flow regimes (i.e., Hyd+) played a less important role in taxonomic temporal β‐diversity than functional temporal β‐diversity. By contrast, the addition of nutrient regimes (i.e., Nut+) increased explained variations in both taxonomic and functional temporal β‐diversity (Figure 3). Regardless of the potential reasons, which warrant further investigations, these results supported our hypothesis that the addition of environmental regimes could dramatically advance our understanding of environment‐biota relationships.

## 4 CONCLUSION AND REMARKS

SER is a promising tool to facilitate the calculation of environmental regimes over a given time period. As a holistic term, it is suitable for any type of environmental or biotic parameters, such as nutrient concentration, pH, conductivity, light, dissolved oxygen, and chlorophyll a. Furthermore, by changing the argument “days_bf,” it is possible to compute environmental regimes over any given time period, such as months or years, as long as the records are measured in a corresponding manner.

Being a completely open‐source tool, it is open for further extension and examination. We envisage that SER is greatly helpful for both basic and applied ecological studies from mesocosm experiments to field surveys. Environmental regimes (e.g., thermal, nutrient, flow), particularly short‐term environmental regimes, can be robust variables in understanding the “community–environment” relationships of different organisms in various ecosystems (e.g., aquatic, forest, terrestrial ecosystems), being complementary predictors for model simulation and prediction. A recent study found that severe changes in the thermal regimes of Austrian rivers under climate change reinforced physiological stress and supported the emergence of diseases for brown trout (Borgwardt et al., 2020). Moreover, exploring responses of different organisms to environmental regime shifts can be used for management and policy‐making. For instance, by exploring the relationships between the occurrence of cyanobacterial blooms and water‐level regimes, management of water‐level can be a potential mitigation strategy for cyanobacterial blooms (Bakker & Hilt, 2016). Particularly, we would like to emphasize SER's potential in experimental biology or mesocosm experiments, which often last for a relatively short period but could have high‐frequency measured data, e.g., temperature and light. High‐frequency data (at 15‐min interval) of light and water temperature were measured in a microcosm study, and the results indicated light and temperature emerged as significant variables on phytoplankton community attributes (Wijewardene, Wu, Hörmann, et al., 2021).

To a broad extent, environmental regimes can be used in other subjects such as social sciences, socioeconomics, and epidemiology. For example, a recent study (Wu, Wen, et al., 2022) found that increasing temperature variability (calculated as the standard deviation of the average of the same and previous days' minimum and maximum temperatures) has caused a higher human heat‐related mortality. Another example showed that a shift in a temperature regime caused by climate changes may facilitate a pathogen's survival, development, and spillover and have an effect on transmission chains. Pandemic forecasting models (such as COVID‐19) were recommended to integrate these effects, alongside human behavior and awareness (Rodó et al., 2021). A third example is about crop yield in relation to weather regimes. Altered temperature and rainfall regimes, such as unusually cool and wet spring, is reducing global production of staples (e.g., rice, wheat), while, by contrast, some more drought‐tolerant crops (e.g., sorghum) have benefited from such changes (Ray et al., 2019). Developing an empirical model linking crop yield to weather regimes may inform local people with proper crops under future climate scenarios.

## AUTHOR CONTRIBUTIONS

**Yi Zou:** Writing – review and editing (equal). **Tenna Riis:** Funding acquisition (supporting); writing – review and editing (equal). **Naicheng Wu:** Conceptualization (lead); funding acquisition (lead); methodology (lead); project administration (equal); visualization (lead); writing – original draft (lead). **Kun Guo:** Data curation (equal); formal analysis (equal); methodology (equal); software (lead); writing – original draft (lead). **Fengzhi He:** Writing – review and editing (equal).

## CONFLICT OF INTEREST STATEMENT

The authors declare no conflict of interest.

## Supporting information


Table S1:

## Data Availability

The SER package can be downloaded from GitHub (https://github.com/kun‐ecology/SER). An online tutorial is available for this package on the same GitHub repository. SER depends on two existing R packages: tidyverse and lubridate.
