# Supplementary material for: SER: An R package to characterize environmental regimes
Source: Ecol Evol. 2023 Mar 12;13(3):e9882. doi: 10.1002/ece3.9882 (PMC10008288; doi:10.1002/ece3.9882)
Supplement: Supplementary file 1 — Table S1: [file ECE3-13-e9882-s001.docx]

Supporting materials

**SER: an R package to characterize environmental regimes**

Naicheng Wu^1*^, Kun Guo^2^*^ǂ^*, Yi Zou^3^, Fengzhi He^4^, Tenna Riis^5^

^1^ Department of Geography and Spatial Information Techniques, Ningbo University, Ningbo 315211, China

^2^ School of Ecological and Environmental Sciences, East China Normal University, 200241 Shanghai, China

^3^ Department of Health and Environmental Sciences, Xi'an Jiaotong-Liverpool University, 215123 Suzhou, China

^4^ Leibniz Institute of Freshwater Ecology and Inland Fisheries, 12587 Berlin, Germany

^5^ Department of Biology, Aarhus University, Ole Worms Allé 1, 8000 Aarhus C, Denmark

*^ǂ^ This author contributed equally as first author.*

*^*^ Corresponding author: Naicheng Wu (naichengwu88@gmail.com)*

Table S1 Detail explanations for the 11 short-period hydrological indices calculated in the example of SER package (derived from (Guo *et al.* 2021)).

| Code | Unit | Definition (N=BetwSamT, i.e, days between two successive sampling dates) | |
| --- | --- | --- | --- |
| **Magnitude of flow events** | |  |  |
| BetwSamT.MA1 | L s^-1^ | Mean of daily average flow values over N days period before sampling date. | |
| BetwSamT.MA2 | L s^-1^ | Median of daily average flow values over N days period before sampling date. | |
| BetwSamT.MA3 | % | Coefficient of variation over N days period before sampling date. | |
| BetwSamT.MA4 |  | Skewness of N days period flows. | |
| **Frequency of flow events** | |  |  |
| BetwSamT.ML1 | days | Frequency of low flow: Number of days during N days period where the magnitude of flow is below a lower threshold. Low flow is defined as the daily mean flow below the 25th percentile of all daily values for the time period 2016-2018. | |
| BetwSamT.MH1 | days | Frequency of high flow: Number of days where the magnitude of flow remains above a higher threshold. High flow is defined as daily mean flow above the 75th percentile of all daily values for the time period 2016-2018. | |
| BetwSamT.EL1 | days | Frequency of extreme low flow: Number of days during N days period where the magnitude of flow remains below a lower threshold. Extreme low flow is defined as daily mean flow below the 10th percentile of all daily values for the time period 2016-2018. | |
| BetwSamT.EH1 | days | Frequency of extreme high flow: Number of days during N days period where the magnitude of flow remains above a higher threshold. Extreme high flow is defined as flow above the 90th percentile of all daily values for the time period 2016-2018. | |
| **Rate of change of flow events** | | | |
| BetwSamT.RC | L s^-1^ | Mean rate of change over N days period before sampling date. | |
| BetwSamT.RH1 | days | Number of days where flow rises over N days period before the sampling date, i.e., the number of days with positive flow change. | |
| BetwSamT.RL1 | days | Number of days where flow declines over N days period before sampling date, i.e., the number of days with negative flow change. | |

References:

Guo, K., Wu, N., Li, W., Baattrup-Pedersen, A. & Riis, T. (2021) Microbial biofilm community dynamics in five lowland streams. *Science of The Total Environment,* **798,** 149169.
